# Automated quantitative assessment of pediatric blunt hepatic trauma by deep learning-based CT volumetry

**DOI:** 10.1186/s40001-022-00943-1

**Published:** 2022-12-26

**Authors:** Shungen Huang, Zhiyong Zhou, Xusheng Qian, Dashuang Li, Wanliang Guo, Yakang Dai

**Affiliations:** 1 Pediatric Surgery, Children’s Hospital of Soochow University, Suzhou, 215025 China; 2 Suzhou Institute of Biomedical Engineering and Technology, Chinese Academy of Sciences, Suzhou, 215163 China; 3 Department of Radiology, Children’s Hospital of Soochow University, Suzhou, 215025 China; 4 School of Biomedical Engineering (Suzhou), Division of Life Sciences and Medicine, University of Science and Technology of China, Hefei, 230026 China

**Keywords:** Pediatric blunt hepatic trauma, Deep learning, Quantitative assessment, Contrast-enhanced CT

## Abstract

**Background:**

To develop an end-to-end deep learning method for automated quantitative assessment of pediatric blunt hepatic trauma based on contrast-enhanced computed tomography (CT).

**Methods:**

This retrospective study included 170 children with blunt hepatic trauma between May 1, 2015, and August 30, 2021, who had undergone contrast-enhanced CT. Both liver parenchyma and liver trauma regions were manually segmented from CT images. Two deep convolutional neural networks (CNNs) were trained on 118 cases between May 1, 2015, and December 31, 2019, for liver segmentation and liver trauma segmentation. Liver volume and trauma volume were automatically calculated based on the segmentation results, and the liver parenchymal disruption index (LPDI) was computed as the ratio of liver trauma volume to liver volume. The segmentation performance was tested on 52 cases between January 1, 2020, and August 30, 2021. Correlation analysis among the LPDI, trauma volume, and the American Association for the Surgery of Trauma (AAST) liver injury grade was performed using the Spearman rank correlation. The performance of severity assessment of pediatric blunt hepatic trauma based on the LPDI and trauma volume was evaluated using receiver operating characteristic (ROC) analysis.

**Results:**

The Dice, precision, and recall of the developed deep learning framework were 94.75, 94.11, and 95.46% in segmenting the liver and 72.91, 72.40, and 76.80% in segmenting the trauma regions. The LPDI and trauma volume were significantly correlated with AAST grade (rho = 0.823 and rho = 0.831, respectively; *p* < 0.001 for both). The area under the ROC curve (AUC) values for the LPDI and trauma volume to distinguish between high-grade and low-grade pediatric blunt hepatic trauma were 0.942 (95% CI, 0.882–1.000) and 0.952 (95% CI, 0.895–1.000), respectively.

**Conclusions:**

The developed end-to-end deep learning method is able to automatically and accurately segment the liver and trauma regions from contrast-enhanced CT images. The automated LDPI and liver trauma volume can act as objective and quantitative indexes to supplement the current AAST grading of pediatric blunt hepatic trauma.

## Introduction

Trauma is the leading cause of death in children and adolescents, with abdominal injuries accounting for 15% to 25% of all trauma cases in children [1, 2]. Closed injury is a common form of abdominal trauma and accounts for about 80%–90% of cases of abdominal trauma [3, 4]. Child's liver is of relatively large size, fragile parenchyma, and rich blood supply, and is one of the most vulnerable solid organs in blunt abdominal trauma. Blunt liver injury is one of the types of injury with the highest mortality rate among solid organ injuries in children [5].

Contrast-enhanced computed tomography (CT) is the gold standard for diagnosing blunt liver trauma in children and can assess the severity of liver trauma [6]. At present, the CT-driven American Association for the Surgery of Trauma (AAST) liver injury grading system is the most widely used grading system for blunt hepatic injury [7]. Early and accurate judgment of the AAST grade of pediatric blunt liver trauma is very important for optimal triage and management, which can improve the success rate of critical cases and avoid overtreatment [8–11].

However, there is significant intra- and inter-observer variability when visually assessing liver trauma based on the AAST grading system [12]. In addition, it is difficult and time-consuming to quantify abnormalities resulting from blunt hepatic injury by visual examination of CT images. For example, the percentage of liver parenchyma disrupted by laceration or intraparenchymal hematoma is one of the main CT imaging criteria for determining the AAST grade. In 2021, Dreizin et al. coined the term ‘liver parenchymal disruption index’ to measure the degree of parenchymal injury, which is abbreviated to LPDI for simplicity [13]. The LPDI is computed as the ratio of liver trauma volume to liver volume, where liver trauma volume and liver volume are conventionally obtained based on manually labeled CT images. Since manual labeling is a tedious and costly task, the LPDI based on manual segmentation would be infeasible in routine clinical practice [14].

Recently, there have been limited attempts to use deep learning [15] for quantitative trauma diagnosis based on CT images. Dreizin et al. utilized a multiscale deep learning algorithm [16] for voxel-wise measurements of liver laceration, and found that the derived LPDI was a significantly independent predictor of major hepatic arterial injury in patients with blunt hepatic injury that underwent CT prior to angiography [13]. To our knowledge, this is the first study to segment and quantify liver trauma on CT using computer vision methods. Farzaneh et al. proposed a deep learning framework for automated detection and quantitative assessment of liver trauma, which could be used as a triage tool and monitor volumetric progression or improvements of the trauma region at multiple time points [14]. However, these studies focused on trauma in adults, and the feasibility of deep learning for quantitative assessment of pediatric blunt hepatic trauma has not been clearly established. Considering that children often suffer different injuries from adults due to their different size, anatomy, and physiology [17], this study developed an end-to-end deep learning method for automated quantitative assessment of pediatric blunt hepatic trauma based on contrast-enhanced CT.

## Materials and methods

### Patients

The institutional review board approved this retrospective study and waived the requirement for informed consent. We retrospectively analyzed the portal venous phase CT images of 170 children from May 2015 to August 2021 in the Children's Hospital of Soochow University. The inclusion criteria were as follows: (1) diagnosis of blunt liver trauma in the Children's Hospital of Soochow University; (2) abdominal contrast-enhanced CT performed within 24 h of admission; (3) grading performed according to the AAST grading system.

### Image acquisition

We performed the enhanced CT examination using the GE optima CT660 scanning equipment (GE, US). The pediatric patient was in a supine position, and the scanning range was from the lower chest to the ischial tuberosity. A non-ionic contrast agent (Omnipac, GE Pharmaceuticals Shanghai) for enhanced CT scan was diluted with normal saline; a total amount of 1.5–2.0 ml/kg was injected in 15–20 s. The flow rate was the total amount divided by the injection time (usually 1.0–1.5 ml/s). The tube voltage was 120 kV; the current was 100 mAs; the slice thickness was 5 mm; the collimation was 40 mm; the pitch was 1 mm; the matrix was 512 × 512; the scanning delay in the arterial phase was 20 s; and the scanning delay in the portal venous phase was 60 s. After scanning, the original CT data and reconstructed coronal and sagittal images were uploaded to the workstation, the images were saved in DICOM format, and the cross-sectional CT images were obtained through the hospital's PACS system.

### Ground-truth labeling

**Fig. 1.**
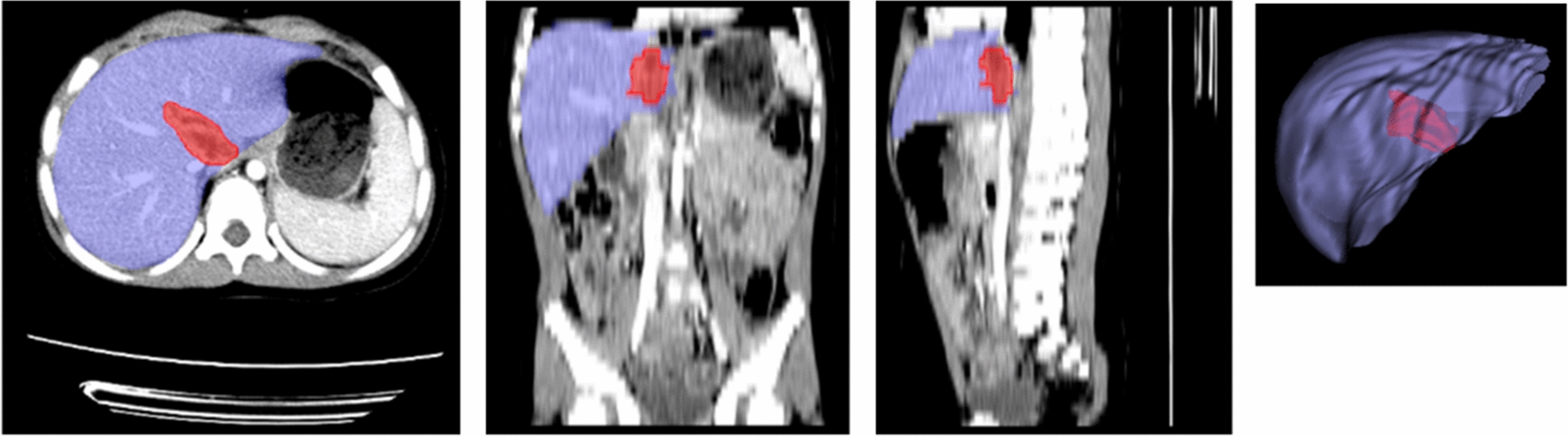
2D and 3D visualization of the manually segmented liver and trauma region

Both liver laceration and intraparenchymal hematoma typically present as regions of low density compared with adjacent unaffected liver parenchyma. To obtain ground-truth labels for all of the 170 pediatric patients, the labeling was performed by two radiologists using an in-house developed software [18]. Each CT scan was manually segmented using a spherical brush tool in a slice-by-slice fashion by a radiologist with 5 years of experience to create binary masks for liver parenchyma and liver trauma regions. Another radiologist with 10 years of experience verified the manual segmentation results. Figure 1 illustrates the manual segmentation results in the two-dimensional (2D) planes (axial, coronal, and sagittal) and three-dimensional (3D) visualization of the segmented liver and trauma region.

### Deep learning-based analysis

**Fig. 2:**
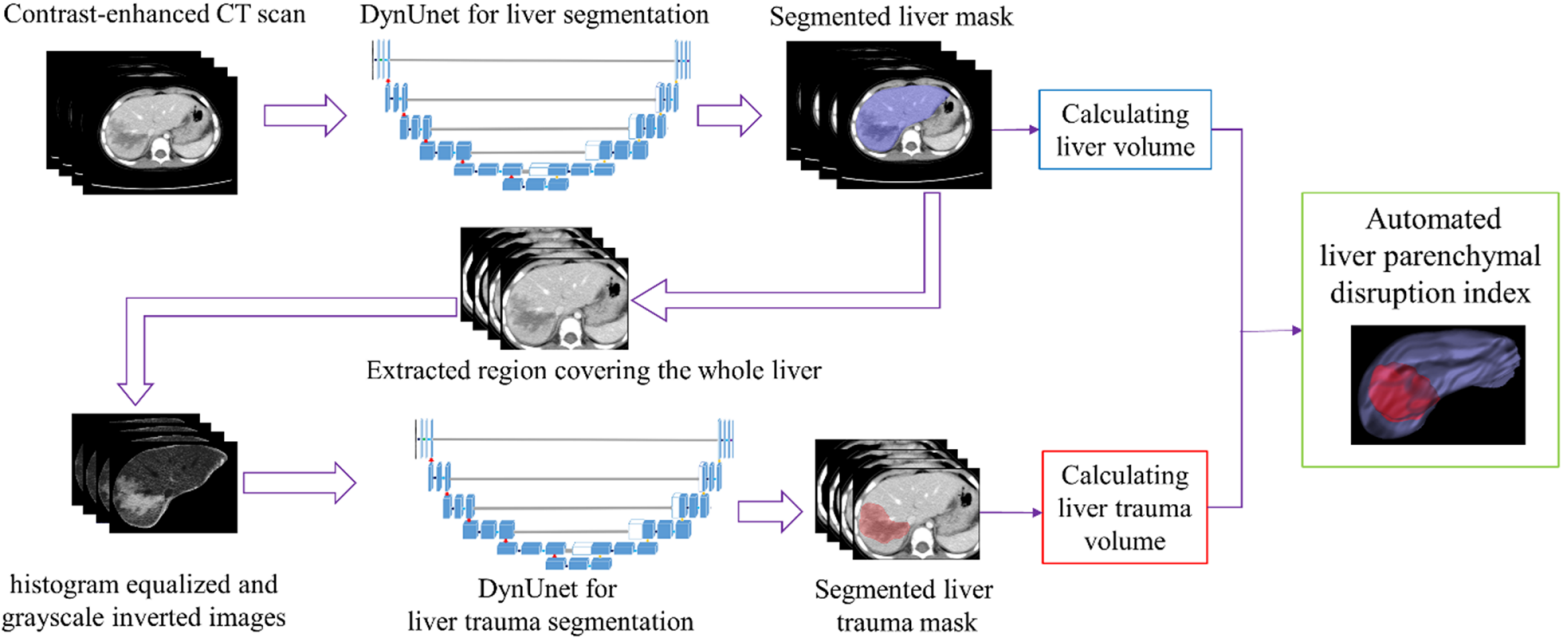
The workflow of the proposed deep learning method for automated detection and quantitative assessment of pediatric blunt hepatic trauma

The proposed end-to-end deep learning framework for automated detection and quantitative assessment of pediatric blunt hepatic trauma is shown in Fig. 2. A liver segmentation model based on the MONAI dynamic UNet^1^ (DynUnet) was first developed to create the initial liver mask for the contrast-enhanced CT scan. The largest 3D connected component of the initial mask was extracted as the predicted liver mask. The liver region was extracted from the CT scans by utilizing the predicted liver mask expanded with a 5-mm-wide margin. To highlight the trauma regions, histogram equalization was performed for the extracted image, and the grayscale was inverted [19]. Based on the grayscale-inverted images, a second dynamic UNet model was constructed to segment the liver trauma regions. Considering that trauma regions were within the liver parenchyma, the predicted trauma masks outside of the predicted liver masks were excluded. After creating the binary masks of the liver and trauma regions, the volumes were computed by multiplying the number of pixels from the binary mask by the unit pixel volume. The unit pixel volume was calculated according to slice spacing and pixel spacing values obtained from CT scan metadata. Finally, the LPDI was calculated according to definition from Dreizin et al. [13] as follows:

1 $${\text{LPDI = }}\frac{{\hat{V}\left( {trauma} \right)}}{{\hat{V}(liver)}} \times 100\% ,$$

where $$\overset{\lower0.5em\hbox{$\smash{\scriptscriptstyle\frown}$}}{V} \left( \cdot \right)$$ represents the estimated volume of a segmentation region.

The employed dynamic UNet is an adaptation of the nnU-Net framework [20], which is currently the most advanced general-purpose approach for medical image segmentation. The dynamic UNet follows the encoder–decoder-based 3D residual UNet architecture [21], which configures two residual blocks per resolution step in both the encoder and the decoder. Each residual block consists of convolution, followed by instance normalization and a leaky ReLU nonlinearity. Downsampling was performed with strided convolutions, and upsampling was implemented as transposed convolution. The initial number of feature maps was set to 32 and doubled (halved) with each downsampling (upsampling) operation. The Adam optimizer [22] wwas used to optimize the network parameters by minimizing the weighted sum of Dice loss and cross-entropy loss [23].

To develop the liver segmentation and liver trauma segmentation models, the MONAI framework2 was used, which is a PyTorch-based framework for deep learning in healthcare imaging [24]. The contrast-enhanced CT scans of 118 pediatric patients between May 1, 2015, and December 31, 2019, were used to train the dynamic UNet with deep supervision. The patient-wise fivefold cross-validation was implemented using the 118 training samples. The contrast-enhanced CT scans of 52 pediatric patients between January 1, 2020, and August 30, 2021, were used to further validate the performance of the developed end-to-end deep learning method.

### Statistical analysis

The performance of the liver segmentation model and the liver trauma segmentation model was evaluated on the validation set using Dice similarity coefficient, recall, precision, and relative volume difference (RVD). The metrics were defined as follows:

2 $${\text{Dice}} = \;\frac{{2 \times \left| {S \cap G} \right|}}{\left| S \right| + \left| G \right|},$$

3 $${\text{Recall}} = \frac{{\left| {S \cup G} \right|}}{\left| G \right|},$$

4 $${\text{Precision}} = \frac{{\left| {S \cup G} \right|}}{\left| S \right|},$$

5 $${\text{RVD}} = \frac{\left| S \right| - \left| G \right|}{{\left| G \right|}},$$

where $G$ and $S$ refer to the manually labeled ground truth and the predicted segmentation mask, respectively. Means with standard deviations (SDs) were calculated for performance evaluation. Pearson’s *r* and intraclass correlation coefficient (ICC) were used to assess correlation and agreement between manual and automated measurements for liver volume, liver trauma volume, and LPDI. Pearson’s *r* ≥ 0.80 and ICC ≥ 0.75 are considered strong correlation and excellent agreement [13]. The Spearman rank correlation coefficient was utilized to investigate whether there were significant correlations between the derived LPDIs and the AAST liver injury grades. A *p* value lower than 0.05 was considered statistically significant. Diagnostic efficiencies of the LPDI and liver trauma volume for severity assessment of pediatric blunt hepatic trauma were evaluated using receiver operating characteristic (ROC) analysis. The optimal cutoff threshold values were determined according to Youden’s index [25]. The analyses were performed using IBM SPSS statistics version 25.0.

## Results

### Patients’ characteristics

A total of 170 pediatric patients with blunt liver trauma were included in our study, including 102 males (60.0%) and 68 females (40.0%). The average age was 65.92 months, and the age range was 8–179 months. According to the AAST classification of liver injury, 9 cases (5.3%) were grade I, 62 cases (36.5%) were grade II, 58 cases (34.1%) were grade III, 35 cases (20.6%) were grade IV, and 6 cases (3.5%) were grade V. Among the causes of injury, there were 116 cases (68.2%) of traffic accidents, 29 cases (17.1%) of falling from heights, and 25 cases (14.7%) of other blunt injuries. On admission, 32 (18.8%) children had shock; the median injury severity score (ISS) was 21 (9–29), and the median Glasgow Coma Scale (GCS) was 15 (15–15). After admission, 74 patients (43.5%) received blood transfusion because of shock or hemoglobin drop. One patient died of brain death after active rescue post-admission. The demographic and clinical characteristics of all patients are listed in Table 1.

**Table 1:** Demographic and clinical characteristics of pediatric patient with blunt hepatic trauma

Characteristics	Total cohort (*n* = 170)
Gender, *n* (%)	
Male	102 (60.0%)
Female	68 (40.0%)
Age (month), mean ± SD	65.92 ± 39.08
AAST grade, *n* (%)	
I	9 (5.3%)
II	62 (36.5%)
III	58 (34.1%)
IV	35 (20.6%)
V	6 (3.5%)
Mechanism of injury, *n* (%)	
Traffic accident	116 (68.2%)
Fall	29 (17.1%)
Other blunt injury	25 (14.7%)
Shock, *n* (%)	32 (18.8%)
ISS value, median (IQR)	21 (9–29)
GCS value, median (IQR)	15 (15–15)
Blood transfusion, *n* (%)	74 (43.5%)
In-hospital survival, *n* (%)	169 (99.4%)

### Liver segmentation

The range of reference liver volumes based on manual liver segmentation is 304.98–1317.1 ml for the training set, and the corresponding mean and standard deviation are 595.49 and 193.80. The liver segmentation performance of the fivefold cross-validation is shown in Table 2, which is averaged over five cross-validation folds and stratified by the AAST grade. The mean Dice, precision, recall, and RVD values are 94.66%, 94.84%, 94.55%, and -0.261%, respectively.

**Table 2:** Liver segmentation performance stratified by AAST grade using fivefold cross-validation on the training set

AAST grade	Dice (%)	Precision (%)	Recall (%)	RVD (%)
I (*n* = 6)	94.70 ± 0.64	95.44 ± 1.37	94.02 ± 2.12	−1.454 ± 3.460
II (*n* = 42)	95.21 ± 0.97	95.15 ± 1.69	95.31 ± 1.23	0.201 ± 2.403
III (*n* = 39)	94.57 ± 1.97	95.10 ± 1.66	94.14 ± 3.57	−0.969 ± 4.310
IV (*n* = 27)	93.86 ± 1.76	93.91 ± 2.56	93.92 ± 3.15	0.115 ± 4.934
V (*n* = 4)	95.02 ± 0.97	94.55 ± 1.12	95.52 ± 1.55	1.031 ± 2.023
Overall (*n* = 118)	94.66 ± 1.61	94.84 ± 1.93	94.55 ± 2.74	−0.261 ± 3.807

**Fig. 3:**
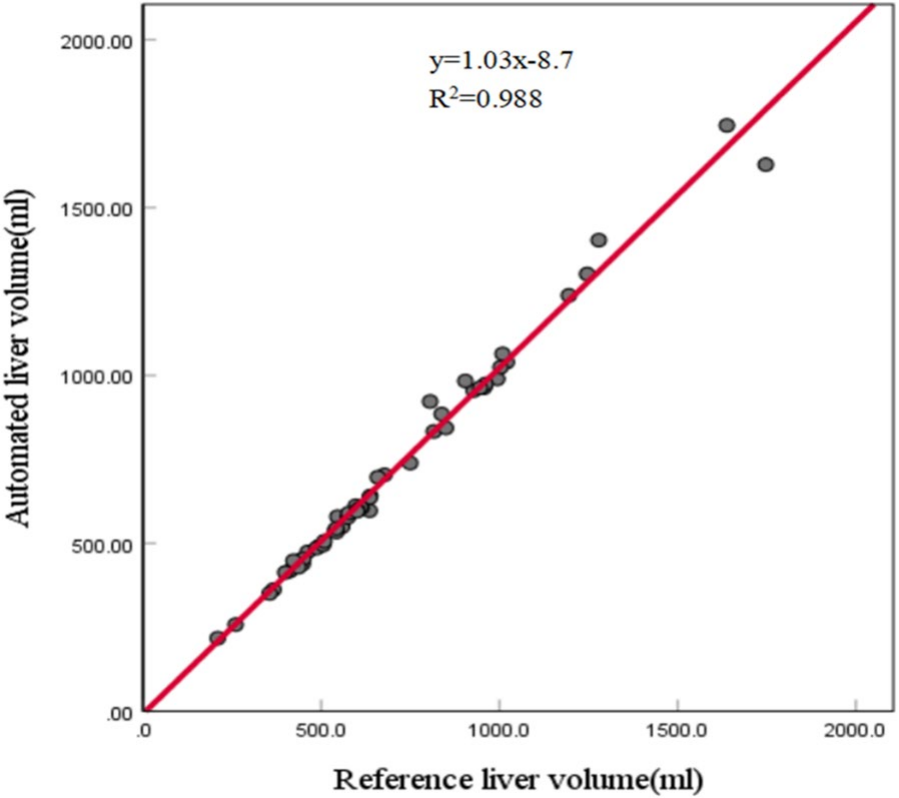
Scatter plot of the reference liver volumes obtained based on manual liver segmentation versus automated liver volumes derived from automated liver segmentation results on the validation set. The linear regression line is shown in red

The range of reference liver volumes of the validation set is 210.30–1738.9 ml, and the corresponding mean and standard deviation are 705.77 and 318.36. The developed liver segmentation model achieved mean Dice, precision, recall, and RVD values of 94.75%, 94.11%, 95.46%, and 1.522% on the validation set, respectively. The detailed performance of liver segmentation stratified by the AAST grade is shown in Table 3. The performance on the cases with the AAST grades IV and V was worse than that on the cases with the AAST grades I–III, which could be due to variations in CT values and contour distortion of the liver caused by laceration or intraparenchymal hematoma. The range of automated liver volumes based on automated liver segmentation is 217.48–1744.4 ml, and the corresponding mean and standard deviation are 719.15 and 330.77. The comparison between reference liver volumes and automated liver volumes of the validation set is shown in Fig. 3. The linear regression yielded a high R^2^ value of 0.988 with *p* < 0.001. Pearson’s *r* and ICC revealed excellent correlation and agreement with values of 0.994 and 0.993, respectively. These results showed that the developed liver segmentation model achieved excellent performance in estimating the liver volume for children with blunt liver trauma based on contrast-enhanced CT.

**Table 3:** Performance of the liver segmentation model on the validation set stratified by the AAST grade

AAST grade	Dice (%)	Precision (%)	Recall (%)	RVD (%)
I (*n* = 3)	95.99 ± 0.38	95.05 ± 2.06	96.99 ± 1.38	2.095 ± 3.651
II (*n* = 20)	94.99 ± 1.43	94.56 ± 2.24	95.46 ± 1.72	1.006 ± 3.060
III (*n* = 19)	95.13 ± 0.85	94.40 ± 1.69	95.89 ± 1.02	1.616 ± 2.469
IV (*n* = 8)	93.54 ± 2.51	92.75 ± 4.52	94.52 ± 3.25	2.172 ± 7.106
V (*n* = 2)	91.86 ± 3.34	90.81 ± 3.05	92.93 ± 3.66	2.328 ± 0.595
Overall (*n* = 52)	94.75 ± 1.68	94.11 ± 2.63	95.46 ± 1.96	1.522 ± 3.650

### Liver trauma segmentation

The range of reference liver trauma volumes based on manual liver trauma segmentation is 2.0459–254.64 ml for the training set, and the corresponding mean and standard deviation are 47.813 and 47.528. The liver trauma segmentation performance of the fivefold cross-validation is shown in Table 4, which is averaged over five cross-validation folds and stratified by the AAST grade. The mean Dice, precision, recall, and RVD values are 72.52%, 79.53%, 70.14%, and -5.686%, respectively.

**Table 4:** Liver trauma segmentation performance stratified by AAST grade using fivefold cross-validation on the training set

AAST grade	Dice (%)	Precision (%)	Recall (%)	RVD (%)
I (*n* = 6)	52.89 ± 34.44	50.29 ± 37.44	59.38 ± 32.01	52.79 ± 66.30
II (*n* = 42)	71.84 ± 16.76	79.86 ± 18.77	69.23 ± 19.20	−2.386 ± 54.04
III (*n* = 39)	74.36 ± 18.30	79.75 ± 17.33	72.21 ± 21.32	−9.320 ± 30.46
IV (*n* = 27)	74.64 ± 22.70	84.82 ± 18.38	70.45 ± 24.28	−18.11 ± 27.45
V (*n* = 4)	76.70 ± 4.76	82.22 ± 8.52	73.57 ± 11.67	−8.765 ± 23.36
Overall (*n* = 118)	72.52 ± 19.89	79.53 ± 20.21	70.14 ± 21.52	−5.686 ± 43.75

**Fig. 4:**
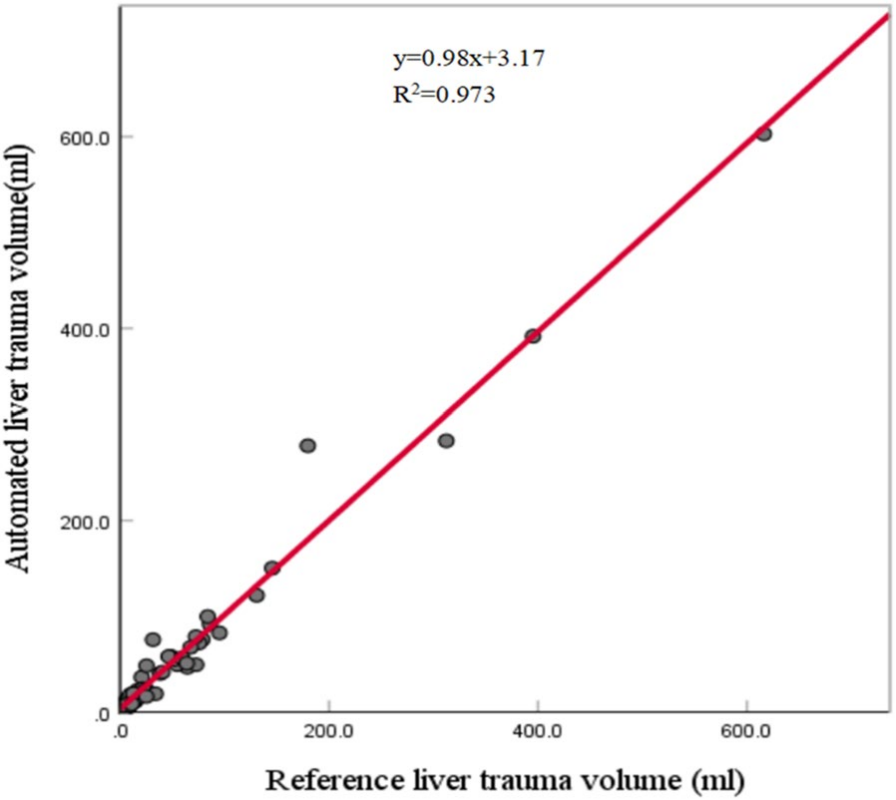
Scatter plot of the reference liver trauma volumes obtained based on manual liver trauma segmentation versus automated liver trauma volumes derived from automated liver trauma segmentation results on the validation set. The linear regression line is shown in red

The range of reference liver trauma volumes of the validation set is 3.3760–616.18 ml, and the corresponding mean and standard deviation are 64.471 and 106.13. The developed liver trauma segmentation model yielded average Dice, precision, and recall values of 72.91%, 72.40%, and 76.80%, respectively, on the validation set. The detailed performance of liver trauma segmentation stratified by the AAST grade is shown in Table 5. Since the number of cases of each AAST grade is small, the standard deviation is very sensitive to extreme values produced by highly erroneous segmentation results. The performance on the cases with the AAST grades I and II was worse than that on the cases with the AAST grades III–V. One major reason of this phenomenon is that it is more difficult for deep learning methods to accurately segment smaller than larger trauma regions [15]. The range of automated liver trauma volumes is 1.7285–602.93 ml, and the corresponding mean and standard deviation are 66.594 and 105.86. The comparison between reference liver trauma volumes obtained based on manual liver trauma segmentation and automated liver trauma volumes obtained based on automated liver trauma segmentation is shown in Fig. 4. The linear regression produced a high R^2^ value of 0.973 with *p* < 0.001. Pearson’s *r* and ICC revealed excellent correlation and agreement with both values equaling 0.986. These results demonstrated that the developed liver trauma segmentation model accurately estimated the liver trauma volume based on contrast-enhanced CT.

**Table 5:** Performance of the liver trauma segmentation model on the validation set stratified by AAST grade

AAST grade	Dice (%)	Precision (%)	Recall (%)	RVD (%)
I (*n* = 3)	52.60 ± 24.02	60.90 ± 22.66	51.05 ± 34.74	-16.88 ± 47.58
II (*n* = 20)	67.06 ± 18.89	65.70 ± 21.79	72.74 ± 22.55	17.48 ± 42.74
III (*n* = 19)	78.42 ± 12.63	77.48 ± 18.75	82.47 ± 9.75	15.21 ± 42.70
IV (*n* = 8)	78.51 ± 6.93	77.48 ± 11.28	80.43 ± 8.16	5.630 ± 22.41
V (*n* = 2)	87.08 ± 4.33	86.59 ± 3.08	87.59 ± 5.60	1.111 ± 2.872
Overall (*n* = 52)	72.91 ± 16.75	72.40 ± 19.65	76.80 ± 18.55	12.22 ± 39.47

### LPDI evaluation

**Fig. 5:**
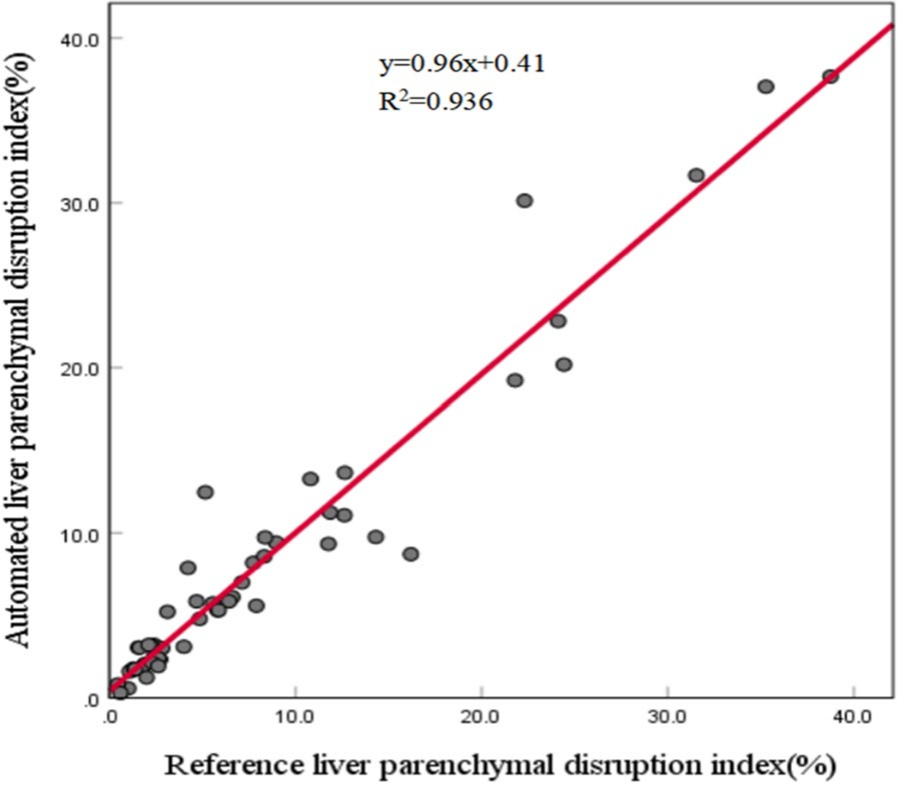
Scatter plot of the reference LPDIs obtained based on manual segmentation versus automated LPDIs derived from automated segmentation results on the validation set. The linear regression line is shown in red

**Fig. 6:**
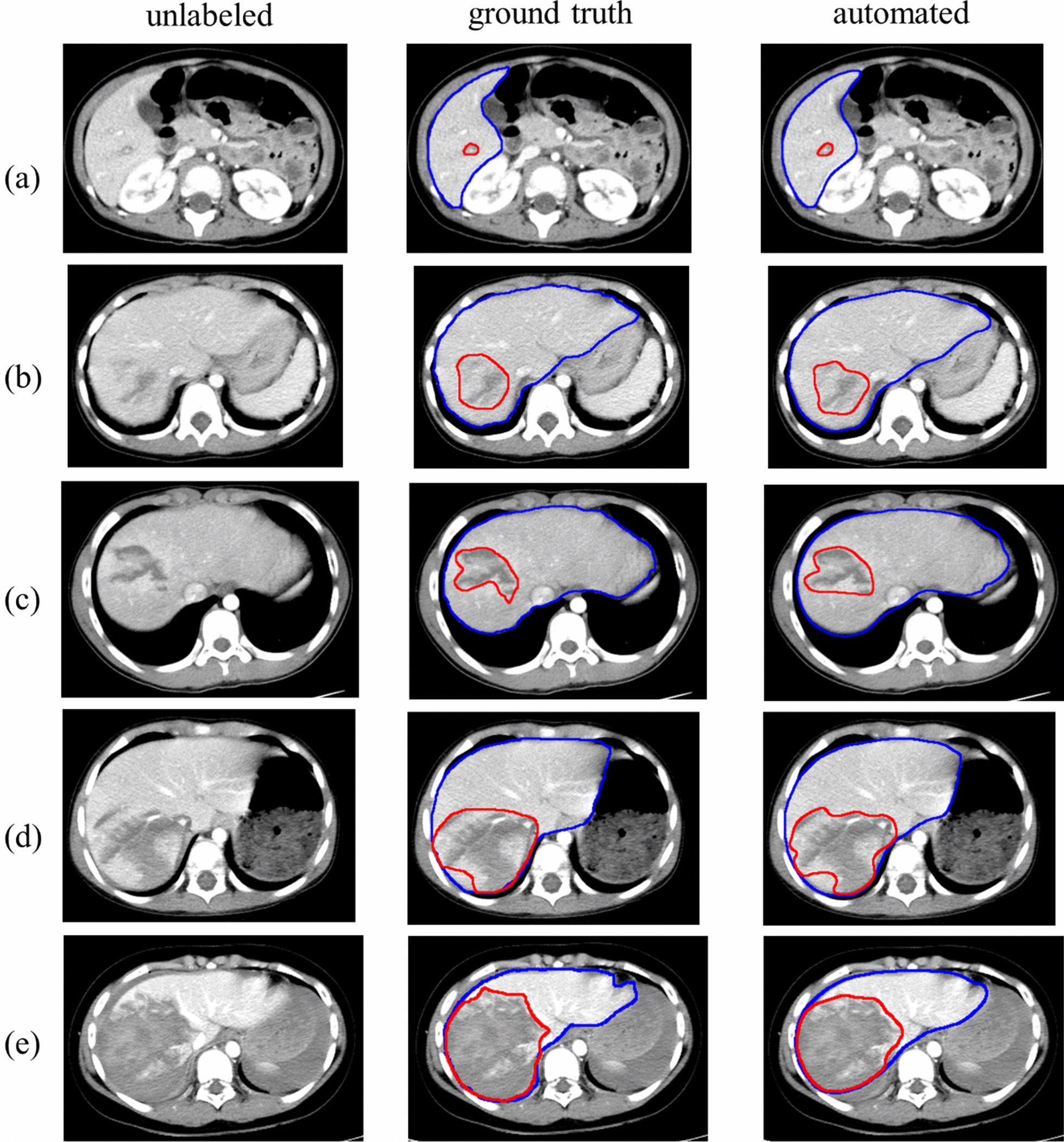
Segmented livers and trauma regions along with ground-truth labels in five pediatric patients with different AAST grades. **a** AAST grade I case with the automated LPDI 3.16% (the reference LPDI is 2.25%). **b** AAST grade II case with the automated LPDI 3.02% (the reference LPDI is 2.94%). **c** AAST grade III case with the automated LPDI 6.11% (the reference LPDI is 6.60%). **d** AAST grade IV case with the automated LPDI 22.82% (the reference LPDI is 24.11%). **e** AAST grade V case with the automated LPDI 37.66% (the reference LPDI is 38.74%). The first column corresponds to the unlabeled CT image; the second column corresponds to the ground-truth labels; and the third column corresponds to the automated segmentation results. The blue line shows the liver contour while the red line represents the contour of trauma regions

The LPDI denotes the percentage of the liver parenchyma affected by blunt traumatic injuries, which can be automatically computed based on the segmented liver parenchyma and trauma regions. The automated liver volumes, trauma volumes, and LPDIs stratified by the AAST grade are shown in Table 6. The automated trauma volumes and LPDIs increased with increasing AAST grade. The Spearman rank correlation analysis revealed that the automated LPDI and liver trauma volume significantly correlated with the AAST grade (rho = 0.823, *p* < 0.001; rho = 0.831, *p* < 0.001, respectively). The comparison between reference LPDIs obtained based on manual segmentation and automated LPDIs obtained based on automated segmentation is shown in Fig. 5. Note that the high deviation of the automated trauma volumes reduces the accuracy of the automated LPDIs, which can be observed from the points falling away from the diagonal line in Fig. 5. Nevertheless, since most of the automated LPDIs are relatively accurate, the linear regression produced a relatively high R^2^ value of 0.936 with *p* < 0.001, which indicates a linear relationship between the reference LPDI and the automated LPDI on the validation set. Pearson’s *r* and ICC also revealed strong correlation and agreement with both values equaling 0.967. Figure 6 illustrates the segmented livers and trauma regions of five pediatric patients with different AAST grades and LPDIs. These results demonstrate that the developed end-to-end deep learning method can provide a relatively accurate estimation of LPDI based on contrast-enhanced CT.

**Table 6:** Automated liver volumes, liver trauma volumes, and LPDIs on the validation set stratified by AAST grade

AAST grade	Liver volume (ml)	Liver trauma volume (ml)	LPDI (%)
I (*n* = 3)	674.73 (183.45)	8.06 (8.21)	1.34 (1.58)
II (*n* = 20)	658.79 (348.11)	17.33 (12.25)	2.90 (1.86)
III (*n* = 19)	724.85 (265.09)	54.05 (24.42)	7.80 (3.18)
IV (*n* = 8)	863.69 (468.40)	190.42 (192.98)	19.52 (10.69)
V (*n* = 2)	757.12 (399.96)	270.91 (170.75)	34.66 (4.24)

### Severity assessment

**Fig. 7:**
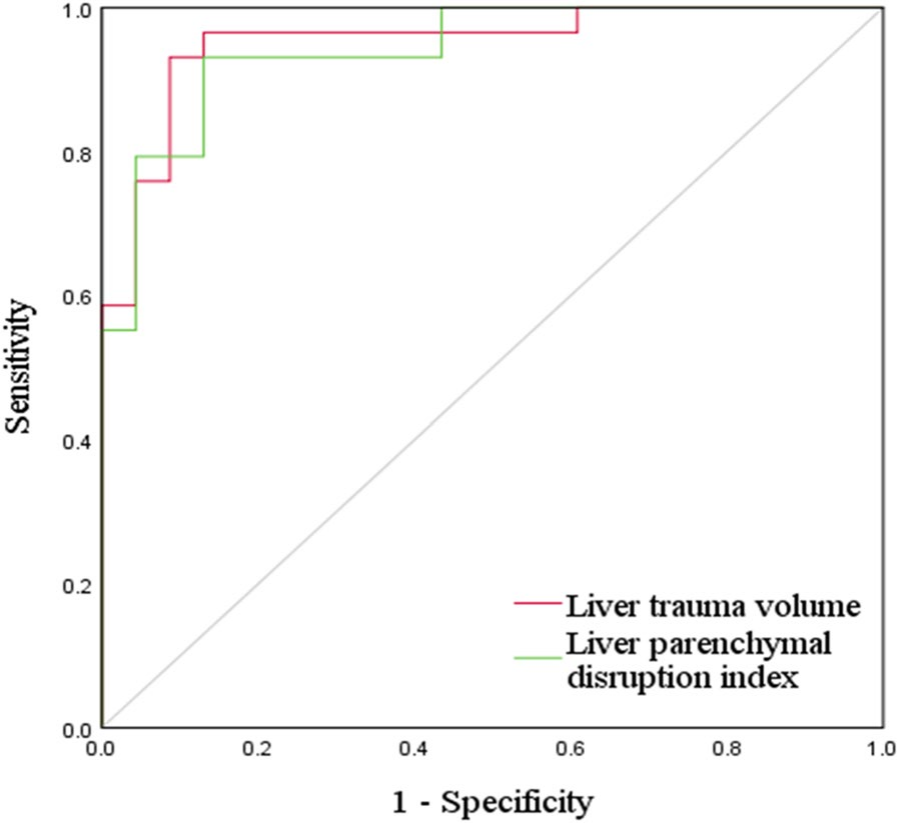
The receiver operating characteristic (ROC) curves of liver trauma volume and LPDI for severity assessment on the validation set

AAST grade I and grade II injuries were grouped as low grade (i.e., low severity), and AAST grades III–V injuries were grouped as high grade (i.e., high severity). The ROC curves corresponding to liver trauma volume and LPDI to discriminate high-grade pediatric blunt hepatic injuries from low-grade injuries are shown in Fig. 7. Table 7 lists the values of AUC, the optimal cutoff values, and the corresponding sensitivity and specificity. The AUC values for the LPDI and trauma volume to distinguish between high-grade and low-grade pediatric blunt hepatic trauma were 0.942 (95% CI, 0.882–1.000) and 0.952 (95% CI, 0.895–1.000), respectively. The optimal cutoff value of liver trauma volume to distinguish high-grade from low-grade injuries was 22.89 ml, and the corresponding sensitivity and specificity were 93.1% and 91.3%, respectively. The optimal cutoff value of LPDI to distinguish high-grade from low-grade injuries was 4.01%, and the corresponding sensitivity and specificity were 93.1% and 87.0%, respectively. These results demonstrate that the developed end-to-end deep learning method can be used for automated quantitative assessment of pediatric blunt hepatic trauma based on contrast-enhanced CT.

**Table 7:** Diagnostic performance of the liver trauma volume and LPDI for severity assessment on the validation set

Index	AUC	Sensitivity (%)	Specificity (%)	Optimal cutoff value
Liver trauma volume	0.952	93.1	91.3	22.89 ml
LPDI	0.942	93.1	87.0	4.01%

## Discussion

This study shows that the developed deep learning method can automatically and accurately segment the liver parenchyma and liver trauma regions from contrast-enhanced CT scans in pediatric blunt liver trauma. The liver trauma volume and LPDI calculated based on the automated segmentation results can be used to objectively and quantitatively evaluate blunt liver trauma in children, and have a high value for the AAST diagnostic grading. At present, the AAST grading system for blunt liver trauma is the gold standard for evaluating the severity of liver trauma injury [26], and it is also the basis for making treatment decisions [3, 11]. However, there are significant differences in the use of the AAST grading system by different physicians to diagnose the severity of liver trauma [27]. In addition, it is necessary to manually analyze a large number of continuous 2D slice images, and it is difficult to perform 3D quantitative analysis intuitively. Not only does it rely on expert experience, and is time-consuming and labor-intensive, but it also has problems such as missed diagnosis, which may lead to delays in diagnosis and treatment planning and affect the prognosis of children. The liver trauma volume and LPDI obtained based on deep learning can help reduce the difference in diagnosis by different physicians, improve the accuracy and efficiency of diagnosis, and provide an objective and quantitative basis for making treatment plans.

In recent years, deep learning has widely been used for computer-assisted detection and diagnosis in medical imaging [28]. However, there are limited studies investigating the utility of deep learning methods for blunt hepatic trauma based on CT. In 2021, Dreizin et al. [13] first introduced the concept of LPDI and used deep learning-based liver parenchymal CT volumetry for predicting major arterial injury after blunt hepatic trauma. They conducted a retrospective study involving 73 adult patients with blunt hepatic injury, and a multiscale attentional network [16] was employed for quantitative visualization of liver laceration on admission contrast-enhanced CT. The average Dice values for liver volume and laceration volume were 95% and 65%, respectively. The derived LPDI was demonstrated to be a significant independent predictor of major hepatic arterial injury in patients with blunt hepatic injury that underwent CT prior to angiography. Farzaneh et al. [14] conducted a retrospective study involving 34 adult patients with evidence of liver trauma and 43 without evidence of liver parenchymal disruption on contrast-enhanced CT. Two U-Net models [29] were developed to segment both liver parenchyma and liver trauma regions using contrast-enhanced CT scans, and the domain knowledge about location and intensity of liver trauma was used to reduce false-positive regions. The average Dice, recall, and precision values were 96.13%, 96.00%, and 96.35% for liver parenchyma and 51.21%, 53.20%, and 56.76% for liver trauma regions. The feasibility of the developed system for both blunt trauma and non-trauma patients showed its potential to be used as a triage tool by rapidly assessing liver injury and its severity. It is noteworthy that the previous studies [14, 15] focused on blunt hepatic trauma in adults only. Compared with adults, children have a relatively large liver with fragile liver parenchyma. Compared with blunt liver trauma in adults, there may be significant differences in the size, shape, and CT attenuation value of the trauma regions in children with blunt liver trauma [30]. In addition, children have a lower systemic blood volume than adults, and are prone to hemorrhagic shock in the early stage. Early and accurate judgment of the severity of pediatric blunt liver trauma is important for making early treatment decisions for children [4, 5]. Therefore, developing an effective deep learning-based method specific for pediatric blunt hepatic trauma is challenging but necessary.

In this study, we aimed to develop an end-to-end deep learning method for automated quantitative assessment of pediatric blunt hepatic trauma and sought to improve model performance by utilizing the state-of-the-art deep learning-based segmentation method nnU-Net [20]. Compared with U-Net and multiscale attentional network [16, 29], which usually requires manual task-specific adaptation, nnU-Net uses a set of readily accessible rules derived from the underlying data to guide the model construction and associated data manipulation, which helps to yield strong generalization characteristics. Consequently, the developed liver segmentation model achieved an average Dice of 94.75%, and the average RVD was 1.522% on the validation set, which is accurate and comparable to the liver segmentation performance in previous studies [13, 14]. Since there are significant variations in the size and shape of injured regions on CT scans, it is difficult for deep learning methods to segment the trauma regions accurately. The developed liver trauma segmentation model achieved an average Dice of 72.91% on the validation set, which is highly competitive with the existing liver trauma segmentation performance (65% in [14] and 51.21% in [15]). Specifically, the average Dice scores of the developed liver trauma segmentation for the AAST grades I, II, III, IV, and V were 52.60%, 67.06%, 78.42%, 78.51%, and 87.08%, respectively. Considering that Dice similarity coefficient is a measure of spatial overlap and highly depends on the relative size of the target [31], the relatively low Dice scores for low-grade pediatric patients are reasonable given the small and irregular trauma regions [13, 14]. Similarly, the average RVD scores for AAST grades I, II, and III were -16.88%, 17.48%, and 15.21%, respectively, which were worse than 5.630% and 1.111% for the AAST grades IV and V, respectively. These results indicate that the developed liver trauma segmentation model can achieve more accurate performance for high-grade pediatric patients. This is favorable in clinical practice since detecting and quantifying a larger and more clinically significant injury can facilitate timely identification of pediatric patients in greatest need of early treatment interventions.

We found that the average liver trauma volume and LPDI calculated by the automatic quantitative calculation increased with increasing AAST grade. The correlation coefficients of liver trauma volume and LPDI with the AAST grade were 0.831 and 0.823, respectively, indicating that liver trauma volume and LPDI are highly positively correlated with the severity of liver trauma. Further ROC curve analysis showed that liver trauma volume and LPDI distinguished low-grade and high-grade blunt liver trauma with AUC of 0.952 and 0.942, respectively; with a sensitivity of 93.1% and 93.1% and a specificity of 91.3% and 87.0%, respectively; and with an optimal cutoff value of 22.89 ml and 4.01%, respectively. These results demonstrate the effectiveness of the deep learning method proposed in our study to automatically obtain two quantitative indicators: liver trauma volume and LPDI. In addition, this method can automatically perform liver trauma region segmentation and 3D modeling, which can assist in the severity assessment of pediatric blunt liver trauma, and is expected to be used for early, rapid, and accurate identification of pediatric patients with severe blunt hepatic injury. In clinical practice, its further combination with clinical laboratory test results, such as hemoglobin content and other indicators, could guide formulation of the best diagnosis and treatment measures to avoid serious complications.

This study has several limitations. First, it was a single center study, and the sample size was small. The generalizability of the deep learning-based segmentation models requires further validation. Second, the retrospective nature of the study could have introduced many forms of bias. Prospective studies with a larger sample size, through collaboration of different centers, are needed. Third, manual labeling using a spherical brush tool lead to weak rather than voxel-wise labeling. The quality of manual labels can be further improved by using a thresholding technique. Finally, this study shows internal validity (correlation with AAST grades) but not clinical validity. The correlation between the deep learning-based CT volumetry and some outcome or relevant intermediate endpoint, such as the need for massive transfusion or failure of non-operative management, should be investigated in future avenues.

## Conclusions

The end-to-end deep learning method developed in this study can automatically and accurately segment the liver and its trauma regions from the enhanced CT images of pediatric blunt liver trauma, perform 3D modeling, and calculate liver trauma volume and LPDI. It can assist in the clinical evaluation of the AAST grade, so as to identify severely injured patients accurately and timely. If the effectiveness and reliability of the developed deep learning method are verified through further multicenter large-sample studies, it may be used as a quantitative analysis tool for automated detection and severity assessment of pediatric blunt hepatic trauma based on contrast-enhanced CT, which is helpful for trauma centers to identify children with severe blunt hepatic injury, so as to improve the success rate of treatment of pediatric blunt liver trauma.

## Data Availability

The datasets used and analyzed during the current study are available from the corresponding author on reasonable request.

## Abbreviations

CT: Computed tomography
CNN: Convolutional neural network
LPDI: Liver parenchymal disruption index
AAST: American Association for the Surgery of Trauma
2D: Two-dimensional
3D: Three-dimensional
RVD: Relative volume difference
SD: Standard deviation
ROC: Receiver operating characteristic

## Footnotes

1 https://docs.monai.io/en/latest/networks.html.
